# Thoughts and Experiences on Chloro-Nitrous Oxide for One Week

**Published:** 1869-07

**Authors:** R. N. Lawrance


DENTAL REGISTER.

VOL. XXIII.] JULY, 1869. [No. 7.

### Original Communications.

### THOUGHTS AND EXPERIENCES ON CHLORO-NITROUS OXIDE FOR ONE WEEK.

BY R. N. LAWRANCE, D. D. S.

The record of the thoughts and experiences of our professional brethren, the many minor points wherein one practitioner differs from others, reports of cases in practice—in short, to "give in our experience" forms to me the most profitable and interesting part of our Dental literature, and I would that more of the brethren would tell us of their successes and failures. We expect that many truths, and familiar ones, must be often repeated. Experiences in every day practice, even if there be nothing startling or novel, are generally eagerly read. I confess that I am always desirous to have my fellow practitioners to give in their experience and I mine, and always feel benefited thereby. But of late, when about to seek for more light, I always ask, "Have you a patent on your ideas, sir." (You are aware, Mr. Editor, it is fashionable now days to patent very small things.) It does seem to me this is not the proper spirit to be exhibited by professional gentlemen, who desire the success and advancement of our noble specialty. Our present standing is due, in a great measure, to the charity and freedom with which scores of great minds have sown broadcast for all the priceless truths they have discovered in developing our specialty. Then let us not wrap the mantle of selfishness about this little microcosm and say, "Patent applied for"—pay me ere I impart the great secret. In looking over the journals of the past few months we find numerous questions agitating the profession from the rubber question down to chloro-nitrous oxyd. An article in the February number of the REGISTER drew my attention to chloro-nitrous oxyd. I proceeded to try its merits by taking an oz. bottle, with a wide mouth, putting in it a piece of sponge; this bottle I attached to the tubing next the gas receiver, the mouth of the bottle opening into the tube just over the entrance to receiver; when the gas began to come over I took ¼ dr. of chloroform and 1 dr. of alcohol, and mixed and put into the bottle; then the gas passed through the sponge and the chloroform vapor mingled with it. On breathing it I found it strongly impregnated with chloroform. I administered it to nine patients in all, and the majority of them complained of after effects, similar to that produced by chloroform. I found that it induced anæsthesia in much less time than nitrous oxyd alone, and also prolonged it, and that recovery was not as speedy as when gas is used.

1st case. A strong, healthy man of bilious temperament. I heard no complaints from him.

2d. A young man of bilious temperament; complained of an intense feeling of internal restlessness and mental images were conjured up.

3d case. An old gentleman of nervous temperament, to whom I had before administered gas. This combination acted very rapidly and the anæsthesia was more profound; showed pallor of the countenance. The cessation of respiratory activity, as announced by stertor, was very sudden, and a slowness of the pulse following, not observed when the patient was under the influence of pure gas alone.

4th case. A young lady of sanguine temperament; passed into the stage of insensibility very quickly; no peculiar changes observed, but she afterward informed me that she was troubled with a "splitting headache," and felt badly for hours after leaving the office.

5th case. Young lady of nervous temperament; came from the office of a neighboring practitioner who failed to extract the tooth desired; suffering intense pain; I applied Phenol Sodique in and about the tooth and waited a few minutes, then proceeded to administer the gas. The patient was very restless and the senses abnormally excited; convulsive movements and mental disturbances continued during the whole period of anæsthesia.

6th case. A lady of some 35 years of age; nervous-sanguine temperament; brought in by her family physician, who had been treating her for neuralgia. She was very much excited when she came, but passed into insensibility with no marked symptoms, more than a degree of wildness and sobbing when returning to consciousness. I saw her in a few days after and she informed me that in an hour or two after leaving the office she was attacked with a congestive chill, that her limbs became cold and lifeless, and her friends had to apply warm applications, friction, &c., to keep her alive.

7th case. A stout hearty man. No trouble or after indications.

8th case. A middle aged lady of nervous-bilious temperament. Afflicted with neuralgia and in an anæmic condition; had been troubled with heart disease for many years; any sudden excitement or labor would bring on the manifestations; she came for the special purpose of having gas administered; I declined to give it, informing her that no anæsthetic should be used in her case; herself and friends thought it would do no harm; my partner was of the opinion we had better try it; I told her I would allow her to breathe three or four inspirations and see how it effected her; she did so, I watching the countenance and pulse closely; there was complete muscular quietude, but a great pallor of the countenance, lips colorless and the pulse lowered rapidly; I stopped the gas; she lay in a sort of stupor; I asked her if she felt any pain or annoying sensations in the throat, lungs or about the heart; said no, but she felt paralyzed and lifeless; thought she could go to sleep very easily. I gave her brandy; she revived and became quite talkative after I had given her two drinks of brandy; I then proceeded to extract the teeth; had no trouble; gave brandy at intervals; she went away and was feeling quite well next day.

9th case. A healthy young man; no trouble arising. This is my last with chloro-nitrous oxyd. I have lost no faith in nitrous oxyd, but do not propose to risk its reputation by allowing it to keep bad company. I have no desire to have an exhibition of *facies hippocratica* before me, and the only way to avoid censure, and, above all, to remain at peace with one's own conscience, is to discard a dangerous agent which puts to trial the fearful issue of life or death.

I would like Prof. Watt or Cutler to inform us if, in the combination of nitrous oxyd gas and chloroform vapor, the mixture is mechanical or chemical.

